# Technical Review of Magnetic Resonance Fingerprinting Applications in Cerebral Physiology

**DOI:** 10.1002/mrm.70216

**Published:** 2025-12-07

**Authors:** Chieh‐Te Lin, Hanzhang Lu, Audrey P. Fan

**Affiliations:** ^1^ Department of Biomedical Engineering University of California, Davis Davis California USA; ^2^ Department of Biomedical Engineering Johns Hopkins University School of Medicine Baltimore Maryland USA; ^3^ The Russell H. Morgan Department of Radiology and Radiological Science Johns Hopkins University School of Medicine Baltimore Maryland USA; ^4^ F. M. Kirby Research Center for Functional Brain Imaging Kennedy Krieger Institute Baltimore Maryland USA; ^5^ Department of Neurology University of California, Davis Sacramento California USA

**Keywords:** cerebral physiology, neurovascular health, physiological magnetic resonance fingerprinting, quantitative MRI, vascular hemodynamics

## Abstract

Magnetic resonance fingerprinting (MRF) enables quantitative MRI by allowing the simultaneous mapping of multiple tissue properties through innovative acquisition and computational methods. This review focuses on the application of MRF techniques to cerebral physiology, emphasizing advancements in vascular imaging and the integration of biophysical modeling. We discuss the principles of MRF, its adaptation to quantify hemodynamic and vascular parameters, and its potential to overcome challenges in mapping vascular‐related parameters. The review categorizes MRF‐based imaging approaches, including MRF‐arterial spin labeling (MRF‐ASL), MR vascular fingerprinting (MRvF), and vascular fluid dynamics‐MRF (VFD‐MRF), highlighting their technical implementations, accuracy, and clinical applications in conditions such as stroke, brain tumors, and cerebrovascular diseases. We also explore the role of machine learning in enhancing dictionary matching and reducing computational time for more accurate and reliable real‐time parameter estimation. The challenges such as low signal‐to‐noise ratios and computational demands are addressed through tailored sequence designs, noise‐resilient dictionaries, and deep learning approaches. This comprehensive review provides a detailed technical framework for advancing the role of MRF in assessing cerebral physiology and its clinical translation.

## 1 Introduction

Cerebral physiology encompasses critical aspects of brain function, including blood flow, oxygenation, and vascular integrity, which are essential for maintaining neural activity and overall brain health. Accurate assessment of these parameters is crucial for diagnosing and managing neurological disorders such as stroke, brain tumors, dementia, and other cerebrovascular diseases [1, 2, 3]. Conventional MRI‐based techniques, such as perfusion‐ and oxygenation‐sensitive imaging, have been widely used to evaluate cerebral physiology but often face trade‐offs between spatial resolution, temporal efficiency, and quantitative accuracy [4]. These limitations not only pose risks to patients, especially in vulnerable populations such as those with compromised health or pediatric patients, but also limit the frequency and applicability of these diagnostic tools in routine clinical practice [5, 6]. The need for safer, non‐invasive imaging modalities that can provide comprehensive and quantitative assessments of cerebral physiology has driven research into advanced MRI techniques.

MRI assessment of cerebral vasculature offers non‐invasive means to quantify cerebral hemodynamics, including dynamic susceptibility contrast (DSC) imaging, arterial spin labeling (ASL), and dynamic contrast‐enhanced (DCE). DSC MRI acquires T_2_*‐weighted images during the passage of a gadolinium bolus to quantify cerebral blood volume (CBV), cerebral blood flow (CBF), and mean transit time (MTT), though its reliance on contrast limits its uses in patients with renal impairment [7]. ASL offers a non‐invasive alternative by using magnetically labeled arterial blood water to measure CBF without contrast, making it ideal for longitudinal and pediatric studies, albeit with a lower signal‐to‐noise (SNR) ratio and resolution [8, 9]. DCE MRI complements these techniques by using T_1_‐weighted imaging to assess blood–brain barrier (BBB) permeability through quantification of parameters like the volume transfer constant [10, 11]. DCE is particularly valuable in evaluating BBB integrity across neurological conditions such as brain tumors, multiple sclerosis, and neurodegenerative diseases [12, 13]. While techniques like DSC, ASL, and DCE each provide valuable insights into specific aspects of cerebral physiology, they are inherently limited in scope, often focusing on isolated parameters such as perfusion and permeability. This compartmentalized approach requires multiple separate acquisitions to obtain a comprehensive physiological profile, leading to increased scan times, potential misregistration between datasets, and challenges in integrating data from different modalities.

Magnetic resonance fingerprinting (MRF) offers a non‐invasive, quantitative, and comprehensive approach to mapping multiple cerebral tissue parameters in a single scan [14]. The technique leverages the random or pseudo‐random variation of imaging parameters such as repetition time (TR) and flip angle (FA) to create unique signal evolutions, or “fingerprints,” for different combinations of tissue properties. This paradigm allows for simultaneous mapping of multiple parameters, such as T_1_ relaxation, T_2_ relaxation, and field inhomogeneity from a single acquisition. Unlike traditional MRI techniques that employ fixed pulse sequences optimized for individual parameters [15], MRF enables the design of acquisition schedules that encode sensitivity to multiple tissue properties within a single sequence. Recent advancements have extended MRF capabilities beyond tissue relaxation properties such as T_1_ and T_2_ relaxation to measure critical physiological parameters related to cerebral hemodynamics to assess cerebrovascular health [16, 17]. This extension holds the promise of extracting multiple hemodynamic and vascular parameters in a single acquisition in a way that conventional methods cannot easily achieve.

### 1.1 Basics of Physiological MRF

Physiological MRF refers to the extension of the fingerprinting concept such that the acquisition strategy and dictionary generation are tailored to quantify parameters of vascular physiology, hemodynamics or microstructure rather than only relaxation rates and field maps. In a standard relaxometry‐based MRF sequence (e.g., inversion recovery balanced steady‐state free precession (IR‐bSSFP) or fast imaging with steady‐state precession (FISP) with varying TR/FA), the pulse sequence is designed so that each tissue's unique T_1_/T_2_ yields a distinct “fingerprint”. In physiological MRF, the sequence likewise uses a variable train of FA and timing but also incorporates pulses or gradient modules to sensitize the signal to vascular or microvascular structures. For example, a flow‐sensitive preparation may include bipolar gradients to encode velocity, while a perfusion‐sensitive MRF acquisition may interleave label/control modules and varying post‐label delays [18]. This means the dictionary must simulate not only relaxation and exchange but also flow, transit time, vascular compartment contributions and possibly macrovascular suppression or labeling efficiency variations. Because these physiological effects modulate the time course of magnetization, the sequence design, TR/FA trains, and labeling/gradient modules all diverge from the “standard” MRF template. Thus, physiological MRF sits conceptually between conventional quantitative MRI and dynamic vascular imaging: it retains the fingerprint‐matching paradigm, but the acquisition is engineered to capture temporal and compartmental modeling of physiology.

**FIGURE 1:**
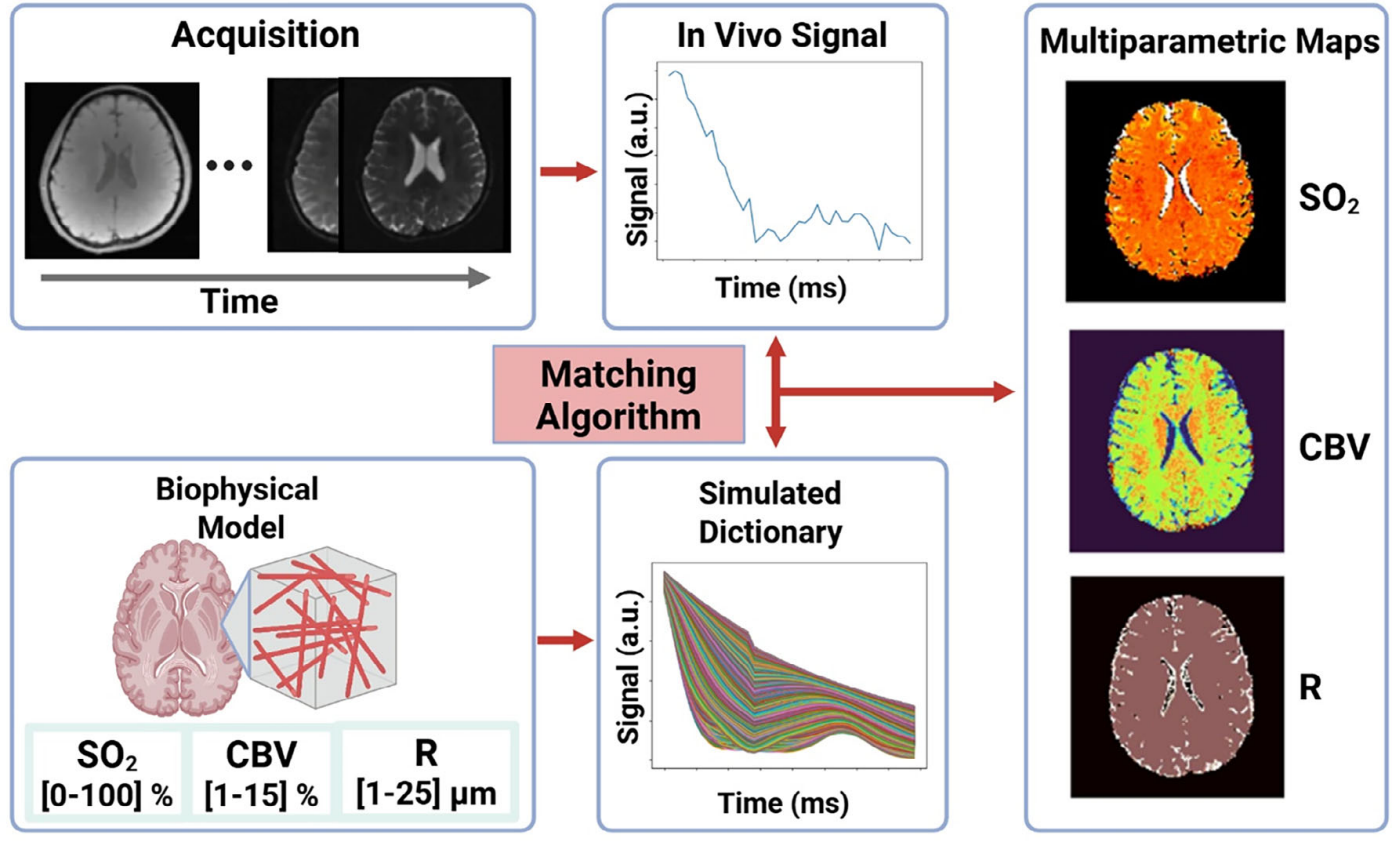
Overview of physiological MRF acquisition and fingerprinting process: In vivo signal evolutions acquired over time are matched to a simulated dictionary generated from biophysical models of vascular parameters (SO_2_, CBV, R). The best‐fitting dictionary entry determines the voxel‐wise multiparametric maps.

As illustrated in Figure 1, the workflow of physiological MRF (demonstrated here by MR vascular fingerprinting (MRvF)) integrates signal acquisition, biophysical modeling, and quantitative parameter mapping. The process begins with the acquisition of in vivo signal evolutions using a multi‐echo sequence such as gradient‐echo sampling of free induction decay and echo (GESFIDE) [19], which captures both free induction decay and spin‐echo components to enhance sensitivity to oxygen saturation (SO_2_), CBV, and vessel radius (R). These time‐resolved signal evolutions are compared with a large precomputed simulated dictionary derived from a biophysical model that spans a range of physiological parameters. A matching algorithm then identifies the best‐fitting dictionary entry for each voxel, producing multiparametric maps that simultaneously retrieve the parameters of interest across the brain. In the physiological MRF, the estimated parameters can be broadly categorized according to their biological relevance, which includes hemodynamic, vascular, oxygenation, and supporting MR properties. Table 1 summarizes these physiological and supporting parameters in physiological MRF, grouping them into functional domains that reflect their role in characterizing cerebral perfusion, vascular architecture, and tissue microenvironment. This integrated approach provides non‐invasive, multi‐parametric quantification of cerebral physiology from a single acquisition for improved efficiency and reduced sensitivity to motion and noise compared to traditional multi‐sequence methods.

This review describes the theory and practical elements of MRF for brain physiology, including simulation, dictionary matching (DM), and the in vivo mapping of cerebral physiological parameters. While a recent review by Coudert et al. [17] has covered key advancements in MRvF, our review provides a distinct technical framework by systematically categorizing the field into three primary applications. We categorize different imaging techniques that leverage MRF to measure cerebrovascular physiology such as (1) ASL, (2) MRvF, and (3) vascular fluid dynamics (VFD) (e.g., DSC) and examine their models and clinical and research applications (Table 1). Finally, the review will address current technical challenges and limitations of MRF and explore potential future directions for expanding its role in cerebral physiology and clinical applications.

**TABLE 1:** Summary of physiological and supporting parameters in physiological MRF. The parameters are grouped into functional categories based on their biological relevance.

Category	Parameter (Abbreviation)	Parameter (Full name)	Units	Physiological significance
Hemodynamics: Blood Flow and Transit	CBF	Cerebral Blood Flow	mL/100 g/min	Measures the rate of blood delivery to brain tissue, which is essential for maintaining neural activity. Its quantification is crucial for diagnosing and managing conditions like stroke and cerebrovascular diseases
	BAT/ATT	Bolus Arrival Time/Arterial Transit Time	ms or s	Represents the time it takes for arterial blood to reach the brain tissue after being labeled. Measuring this parameter is critical for identifying delayed perfusion, a key feature in pathologies such as stroke and Moyamoya disease
Vascular Architecture and Volume	CBV/rCBV	Cerebral Blood Volume/relative Cerebral Blood Volume	%	Quantifies the volume of blood within a given amount of brain tissue. It provides information on vascular density and microvascular architecture, which can be altered in brain tumors, stroke, and cerebrovascular diseases
	R	Vessel Radius	μm	Indicates the average size of microvessels. Changes in vessel size signify vascular remodeling or dysfunction, which are characteristic features of atherosclerosis and tumor angiogenesis
Vascular Function and Permeability	K_trans_/Permeability	Volume Transfer Constant	s^−1^	Reflects the rate at which a substance (e.g., contrast agent) moves from blood vessels into the extravascular space. It is a key indicator of blood–brain barrier (BBB) integrity and is used to assess BBB disruption in conditions like brain tumors and multiple sclerosis
	τb	Intravascular Water Residence Time	ms	Measures the average time a water molecule stays within a blood vessel before exchanging across the blood–brain barrier. It serves as a non‐contrast marker for BBB water permeability, which may be altered in early stages of neurodegenerative diseases
	CVR	Cerebrovascular Reactivity	Not specific	Assesses the ability of cerebral blood vessels to change diameter in response to a vasodilatory stimulus. It is a measure of vascular health and functional reserve, providing insight into the brain's ability to regulate its own blood supply
Blood Oxygenation	SO_2_	Oxygen Saturation	%	Measures the percentage of oxygenated hemoglobin in the blood. It reflects the balance between oxygen supply and consumption in the brain, offering critical insights into tissue metabolism and oxygenation status in conditions like stroke and brain tumors
Supporting MR Parameters	T_1_	Longitudinal Relaxation Time	ms	An intrinsic magnetic property of tissue influenced by the local molecular environment. Pathological changes, such as edema in stroke lesions, can cause prolonged T_1_ times, making it a useful biomarker for tissue characterization
	T_2_	Transverse Relaxation Time	ms	An intrinsic tissue property that reflects the tissue's molecular environment. Alterations, such as increased T_2_ values, can serve as early biomarkers for pathological features like neuronal loss, demyelination, or gliosis in neurodegenerative diseases
	B_1_/B_1_^+^	Radiofrequency Transmit Field/Relative Transmit Field	%	A technical parameter measuring the spatial uniformity of the radiofrequency field used for excitation. Correcting for B_1_^+^ inhomogeneities is crucial for ensuring the accuracy and reproducibility of quantitative physiological maps derived from MRF

*Note*: This table provides a comprehensive overview of the key parameters discussed in this review. The parameters are grouped into functional categories based on their biological relevance.

## 2 MRF‐Arterial Spin Labeling (MRF‐ASL)

### 2.1 Physiological Parameters and Acquisition

Traditional ASL methods for non‐invasive cerebral perfusion imaging are limited by low SNR, motion sensitivity, and the need for multiple acquisitions to capture different hemodynamic parameters such as CBF, BAT, and tissue T_1_ [20]. These limitations are particularly problematic in pathological conditions where perfusion is altered or delayed, such as in stroke or Moyamoya disease [21]. MRF‐ASL addresses some of these challenges by integrating the MRF framework with ASL for allowing simultaneous quantification of CBF, BAT, T_1_, and B_1_ in a single, efficient scan [9, 22] (Table 2). The technique uses pseudorandomized labeling durations and control conditions to scan the acquisition of hundreds of effective delay times within a single session for capturing the full kinetic curve of labeled blood water with improved temporal resolution compared to conventional multi‐delay ASL.

**TABLE 2:** Quantitative scanning strategies in cerebral physiology.

	Pulse sequence	Acquisition strategies	Dictionary generation	Quantitative metrics	Matching strategies
MRF‐arterial spin labeling (ASL)					
Wright et al. [23]	Pseudo‐continuous ASL	Multi‐band, multi‐slice ASL sequence with randomized labeling and control pulses with minimum value of PLD	T_1_ [500, 3500] ms B_1_ [−20, 20] % CBV [0.1, 3] [50, 100] % ATT [0.5, 2] s CBF [0, 100] mL/100 g/min BAT [0.5, 3] s	T_1_, B_1_, CBV, ATT, CBF, and BAT	Inner product
Lahiri et al. [24]	Pseudo‐continuous ASL	Pseudorandomized equal numbers of labeled, controlled or without RF pulses	6 × 10^6^ entries T_1_ [330, 3330] ms FA [48°, 112°] CBVa [0, 0.015] CBF [0, 90] mL/100 g/min BAT [0.3, 3] s MTR [0, 0.03] s^−1^	T_1_, FA, CBVa, CBF, BAT, and MTR	Inner product and artificial neural network
Su et al. [25]	Customed MRF‐ASL [22] sequence and gradient echo EPI for DSC scan	Multi‐band, multi‐slice ASL sequence with randomized TRs and labeling orders Gradient echo EPI scan	One compartment model: T_1_ [500, 5000] ms B_1_^+^ [60, 110] % CBF [3, 150] mL/100 g/min Tissue BAT [0.3, 2] s Two compartment model: T_1_ [500, 5000] ms B_1_^+^ [60, 110] % CBF [6, 150] mL/100 g/min Tissue BAT [0.3, 2] s Arterial BAT [0.2, 1] s CBV [0, 5] % Passing‐through blood travel time [200, 1500] ms	One compartment model: T_1_, B_1_^+^, CBF, and BAT Two compartment model: T_1_, B_1_^+^, CBF, tissue BAT, arterial BAT, and passing‐through blood travel time	Inner product
Fan et al. [26]	Customed MRF‐ASL sequence [22]	Multi‐band, multi‐slice ASL sequence with randomized TRs and labeling orders	One compartment model: T_1_ [500, 5000] ms B_1_^+^ [60, 110] % CBF [3, 150] mL/100 g/min Tissue BAT [0.3, 3] s Two compartment model: T_1_ [500, 5000] ms B_1_^+^ [60, 110] % CBF [6, 150] mL/100 g/min Tissue BAT [0.2, 3] s Arterial BAT [0.2, 1] s CBV [0, 5] % Passing‐through blood travel time [200, 1500] ms	One compartment model: T_1_, B_1_^+^, CBF, and BAT Two compartment model: T_1_, B_1_^+^, CBF, tissue BAT, arterial BAT, and passing‐through blood travel time	Artificial neural network
MR vascular fingerprinting (MRvF)					
Christen et al. [16]	2D GESFIDE [19]	Acquisition before and after contrast injection of ferumoxytol with gradient and spin echo signal of 40 echoes with SE = 100 ms	52 920 entries SO_2_ [0, 100] % CBV [0.5, 25] % R [1, 25] μm	SO_2_, CBV, and R	Inner product
Pouliot et al. [27]	2D GESFIDE	Detailed capture of signal evolutions in mouse brain vascular structures before and after superparamagnetic iron oxide nanoparticle injection	SO_2_ [0, 100] % CBV [2, 9] % R [6, 21] μm	SO_2_, CBV, and R	Inner product
Wheeler et al. [28]	SAGE [29, 30]	Acquisition without contrast and using both magnitude and phase information	64 000 entries SO_2_ [0, 100] % CBV [0, 25] % R [2, 25] μm	SO_2_, CBV, and R	Inner product
Delphin et al. [31]	2D GESFIDE	Acquisition before and after contrast injection with gradient and spin echo signal	SO_2_ [35, 90] % CBV [1, 10] % R [2, 10] μm	SO_2_, CBV, and R	Statistical learning
MRF‐vascular fluid dynamics (VFD)					
MacAskill et al. [32]	Customed T_1_‐only sequence	Multiple T_1_‐preparation lobes (adibatic inversion pulses at TI = 20 ms and TI = 500 ms), followed by 3 sequential MRF blocks each with 240 spiral‐FISP images (720 images total per repetition) with a single highly undersampled spiral interleaf (*R* = 48), TR = 10 ms, TE = 2 ms	T_1_ [not specified] T_2_ [2, 602] ms	T_1_, RK^tran^, k_ep_	Inner product
Gu et al. [33]	Balanced steady‐state free precession imaging (bSSFP) and fast imaging with steady‐state free precession (FISP)	Spiral trajectory with significant undersampling to achieve high temporal resolution for DCE‐MRI	T_1_ [100, 4000] ms T_2_ [5, 400] ms Off resonance frequency [−80, +80] Hz	T_1_ and T_2_	Inner product
Venugopal et al. [34]	2D hybrid EPI	Combined spin‐echo and gradient‐echo sequence to capture dynamic susceptibility changes of gadolinium‐based contrast	rCBV [0.5, 10] % R [5, 150] μm	rCBV, and R	Inner product
Thomson et al. [35]	T_1_‐fast field echo	Non‐contrast MRF with sinusoidal flip angle variation optimized for BBB‐related water exchange properties	Blood T_1_ [1500, 1900] ms Tissue T_1_ [600, 2000] ms CBV [1, 10] % τ_b_ [200, 1600] ms B_1_^+^ [0.7, 1.2]	Blood T_1_, tissue T_1_, CBV, τ_b_, and B_1_^+^	Inner product
Van Dorth et al. [36]	Hybrid Echo‐Planar Imaging combining GRE and SE readouts	Two‐bolus DSC‐MRI without contrast preload; GRE and SE acquisition optimized for permeability and vessel radius sensitivity	Permeability [0, 0.0060] s^−1^ R [5, 139] μm rCBV [0.5, 9.2] %	Permeability, R, and rCBV	Inner product

Abbreviations: ATT, arterial time‐to‐peak; CBVa, cerebral blood volume fraction; EPI, echo planar imaging; MTR, magnetization transfer rate; pCASL, pseudo‐continuous arterial spin labeling; rCBV, relative cerebral blood volume; SAGE, combined spin‐ and gradient‐echo.

**FIGURE 2:**
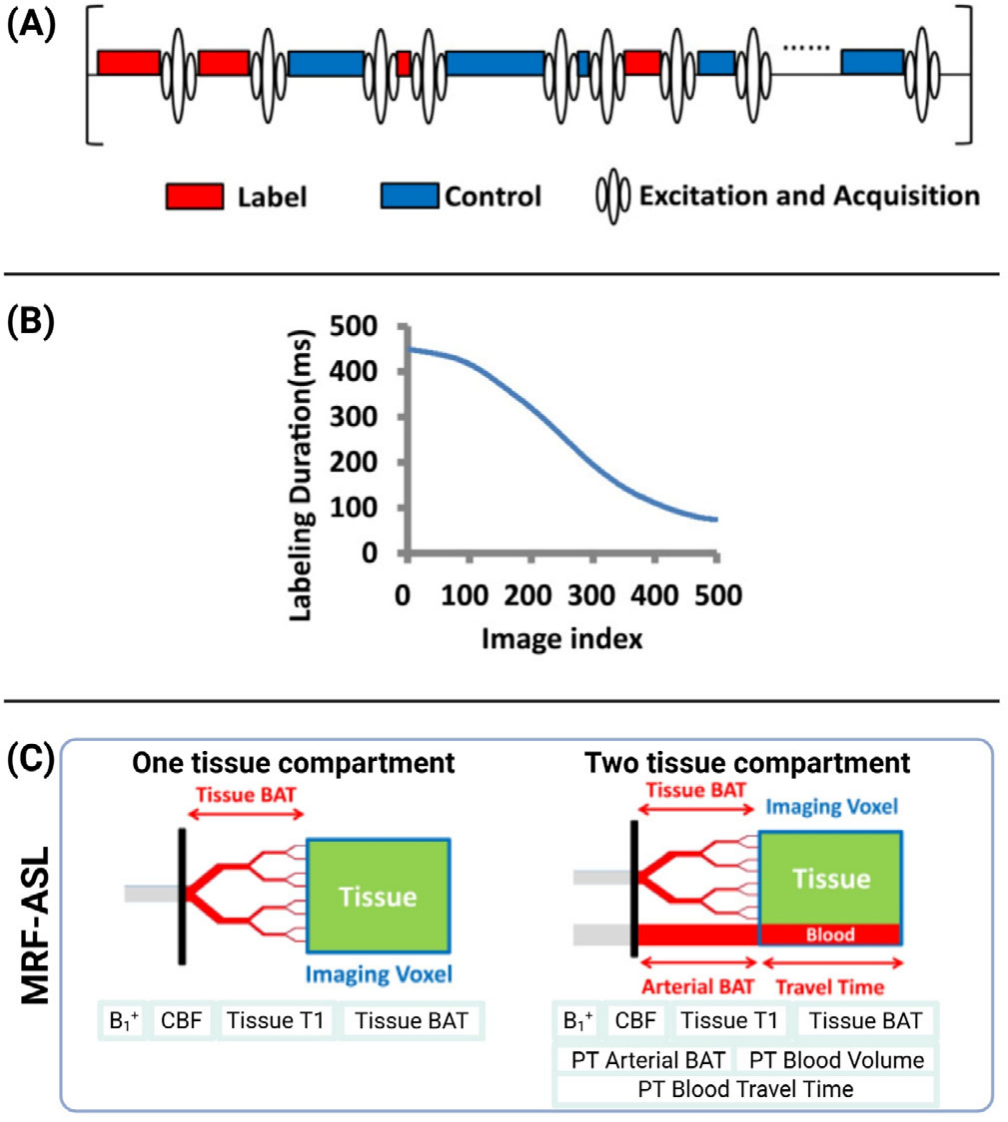
Schematic of MRF‐ASL acquisition and modeling (adapted from Su et al. [22], *Magn Reson Med* 2023). (A) Pseudorandomized label (red) and control (blue) blocks interleaved with excitation/acquisition periods. (B) Variable labeling durations across successive images to sample a wide range of post‐label delays. (C) One‐ and two‐compartment kinetic models used in dictionary generation: The one‐compartment model estimates B_1_^+^, CBF, T_1_, and tissue BAT; the two‐compartment model extends this to include arterial BAT, blood volume, and travel time, providing a richer representation of perfusion dynamics.

As illustrated in Figure 2, MRF‐ASL combines a pseudorandomized label‐control sequence design (Figure 2A) and variable labeling durations (Figure 2B) to encode perfusion kinetics across a broad range of blood transit times [22]. The dynamic labeling strategy yields a unique signal evolution for each voxel, which is compared against a precomputed dictionary based on one‐ or two‐compartment hemodynamic models (Figure 2C). The one‐compartment model estimates parameters such as B_1_^+^, CBF, T_1_, and tissue BAT, whereas the two‐compartment model further accounts for arterial arrival and blood‐volume effects, enabling simultaneous estimation of arterial BAT, perfusion‐territory (PT) blood volume, and blood‐travel time. This modeling enhances physiological specificity and improves the accuracy of perfusion and transit‐time quantification.

While convolutional ASL acquisitions can often handle a few time points corrupted by motion by excluding those control/label pairs during post‐processing, the fingerprinting paradigm of MRF‐ASL presents different constraints. Because the entire time course of pulses and signal evolution is used for dictionary matching, a motion event during the acquisition may distort the fingerprint trajectory and compromise the match quality, potentially introducing bias or blurring of derived parametric maps. Although the inherent redundancy of the fingerprint evolution can afford some resilience, motion perturbations (e.g., bulk head movement or through‐slice shifts) may degrade the precision or accuracy of CBF, transit time, T_1_ and other quantified parameters. Yu et al. showed that motion, off‐resonance and B_1_ inhomogeneity remain important contributors to error in parameter estimation [37].

The acquisition strategy of MRF‐ASL samples the perfusion kinetic curve at hundreds of effective delay times, as opposed to the handful of delayed times used in standard multi‐delay ASL, thus capturing richer temporal information [23]. The need to optimize this scan design for sensitivity to multiple perfusion‐related parameters has led to the adoption of mathematical frameworks such as the Cramer‐Rao Lower Bound (CRLB) for sequence optimization [24]. Machine learning, particularly artificial neural network (ANN), has been introduced for MRF‐ASL to accelerate and improve the accuracy of parameter estimation, addressing computational bottlenecks and enhancing robustness in low‐SNR settings [26, 38]. Clinical studies have demonstrated that MRF‐ASL can detect prolonged BAT with preserved or elevated CBF in Moyamoya disease [39] and effectively differentiate lesion and perilesional tissues in stroke [22], with strong correlations to clinical severity scores. These findings highlight MRF‐ASL's potential to provide clinically relevant hemodynamic information in a single, efficient scan.

### 2.2 Dictionary Simulation

The core of MRF‐ASL Bloch simulations relies on compartment models that account for the dynamics of labeled arterial blood water in different tissue types, tissue relaxation, and field inhomogeneities [18] (Figure 2). In a one‐compartment model, every voxel is treated as homogeneous tissue, such that once the radiofrequency inversion pulse tags the inflowing blood, the labeled spins are assumed to exchange instantaneously with tissue water. Each entry in the dictionary represents a unique combination of parameters (e.g., CBF, BAT, tissue T_1_, and B_1_) that lump arterial transport and capillary exchange together. Another strategy is the two‐compartment model that tracks an explicit arterial or peri‐vascular space in addition to the tissue space. The fingerprint simulation follows two coupled magnetization pools that involve an arterial compartment receiving the inverted bolus first and a tissue compartment that the bolus enters only after a finite transit time. With the added second compartment, in addition to simulating CBF, BAT, tissue T_1_, and B_1_, the dictionary now spans the arterial fraction of cerebral blood volume (CBV_a_), a distinct arterial BAT, and the tissue BAT that describe the travel time of blood through the voxel.

Recent studies have advanced dictionary simulation by incorporating more realistic models of tissue and vascular compartments and by using machine learning to accelerate the matching process. For example, Lahiri et al. used the CRLB framework to guide the design of MRF‐ASL sequences that are optimally sensitive to CBF and BAT, ensuring sufficient variability in labeling durations and post‐label delays to encode distinct temporal features for parameter separation [24]. Typically, thousands to tens of thousands of signal evolutions are simulated to span the expected physiological ranges of CBF, BAT, and T_1_, forming a comprehensive dictionary for subsequent matching.

### 2.3 In Vivo Mapping of Cerebral Vascular Parameters

A major strength of MRF‐ASL is its capacity for simultaneous in vivo quantification of multiple hemodynamic and tissue parameters from a single scan. Early work by Wright et al. established the quantitative potential of MRF‐ASL, demonstrating precise quantification of CBF, arterial BAT, and tissue BAT within a 2.5‐min acquisition. Their Bland–Altman analysis indicated good agreement between MRF‐ASL derived CBF and standard pseudo‐continuous ASL (pCASL) estimates.

The computational demands of matching acquired signals to large dictionaries initially posed a barrier to widespread adoption of MRF‐ASL. However, the integration of machine learning has been transformative, particularly ANN and deep learning. Lahiri et al. pioneered the use of ANN matching strategies, which substantially reduced the computational burden associated with traditional DM and facilitated faster, more accurate parametric mapping. Building on this, Fan et al. incorporated multi‐band imaging with ANN‐based reconstruction, which demonstrated high sensitivity to variations in CBF and BAT while drastically reducing data analysis time from hours (with DM) to mere seconds (with ANN). As shown in Figure 8 from Fan et al., it clearly illustrates the reduction in noise and artifacts in ANN‐derived parametric maps compared to those generated by DM. Similarly, Zhang et al. developed DeepMARS [38], a deep‐learning‐based reconstruction method for MRF‐ASL, which achieved a dramatic reduction in computation time per voxel (less than 0.5 ms versus over 4 s for DM) and concurrently improved the reproducibility of the estimated parameters. Beyond speed, such machine learning approaches enhance map quality by reducing artifacts compared to traditional DM.

### 2.4 Reproducibility

The reliability of quantitative imaging techniques is paramount for their application in longitudinal studies and as clinical biomarkers. Su et al. conducted test–retest scans with an average interval of 20 ± 19 days [25]. They reported high spatial correlation coefficients across several brain regions: B_1_^+^ (0.997), T_1_ (0.962), CBF (0.746), and BAT (0.863). It is noteworthy, however, that the reported reproducibility can vary between different parameters derived from the same MRF‐ASL scan. Parameters estimated with higher intrinsic SNR or less dependence on complex modeling, such as B_1_, may inherently exhibit greater reproducibility compared to parameters like CBF and BAT, which rely on fitting kinetic models to the inherently noisy and complex ASL signal. This implies that users should be cognizant of parameter‐specific confidence intervals when interpreting MRF‐ASL results. An additional benchmark for reproducibility in advanced multiparametric mapping is provided by Fan et al. [40], extending the fingerprinting framework to estimate perfusion parameters in a single acquisition. The authors report very tight reproducibility for ADC, T_2_*, T_1_, and B_1_^+^ (voxel‐wise coefficient of variation, CoV < 5%) and higher variability for perfusion parameters (ATT CoV = 16% ± 3%; CBF CoV = 25% ± 9%). These findings align with the pattern seen in other ASL‐based techniques: parameters with higher intrinsic SNR or simpler model dependence show better reproducibility, whereas those relying on kinetic modeling (CBF/ATT) are more variable.

The reconstruction algorithm itself plays a critical role in the reproducibility of MRF‐ASL parameters. The work by Zhang et al. quantified deep‐learning‐based reconstruction (DeepMARS) reproducibility using the coefficient of determination (R^2^) between its outputs and conventional dictionary matching, as well as the intraclass correlation coefficient (ICC) across repeated scans. Their results showed higher R^2^ and significantly improved ICC for parameters including BAT in the single‐compartment model and CBF in the two‐compartment model in Moyamoya patients. This indicates that advancements in reconstruction methodologies can effectively mitigate variability and enhance the reliability of MRF‐ASL derived maps.

## 3 MR Vascular Fingerprinting (MRvF)

### 3.1 Physiological Parameters and Acquisition

MRvF extends the MRF framework to characterize microvascular properties such as CBV, R, and SO_2_ [16]. Traditional methods like DSC MRI to assess CBV and vessel size imaging (VSI) are limited by their reliance on exogenous contrast agents, partial volume effects, and inability to simultaneously quantify multiple vascular parameters such as oxygenation [41]. MRvF overcomes these limitations by using advanced pulse sequences and biophysical modeling to encode vascular parameters into the MR signal evolution for simultaneous multiparametric mapping. The conventional approach begins with multi‐echo acquisition data, typically using GESFIDE [19], or its variants such as gradient echo sampling of spin echo (GESSE) [42], spin and gradient echo (SAGE) [43], and other quantitative BOLD (qBOLD) sequences that are sensitive to susceptibility and diffusion effects. The dictionary employs Monte Carlo simulations within virtual voxels composed of either idealized cylinders or realistic 3D microvascular reconstructions.

**FIGURE 3:**
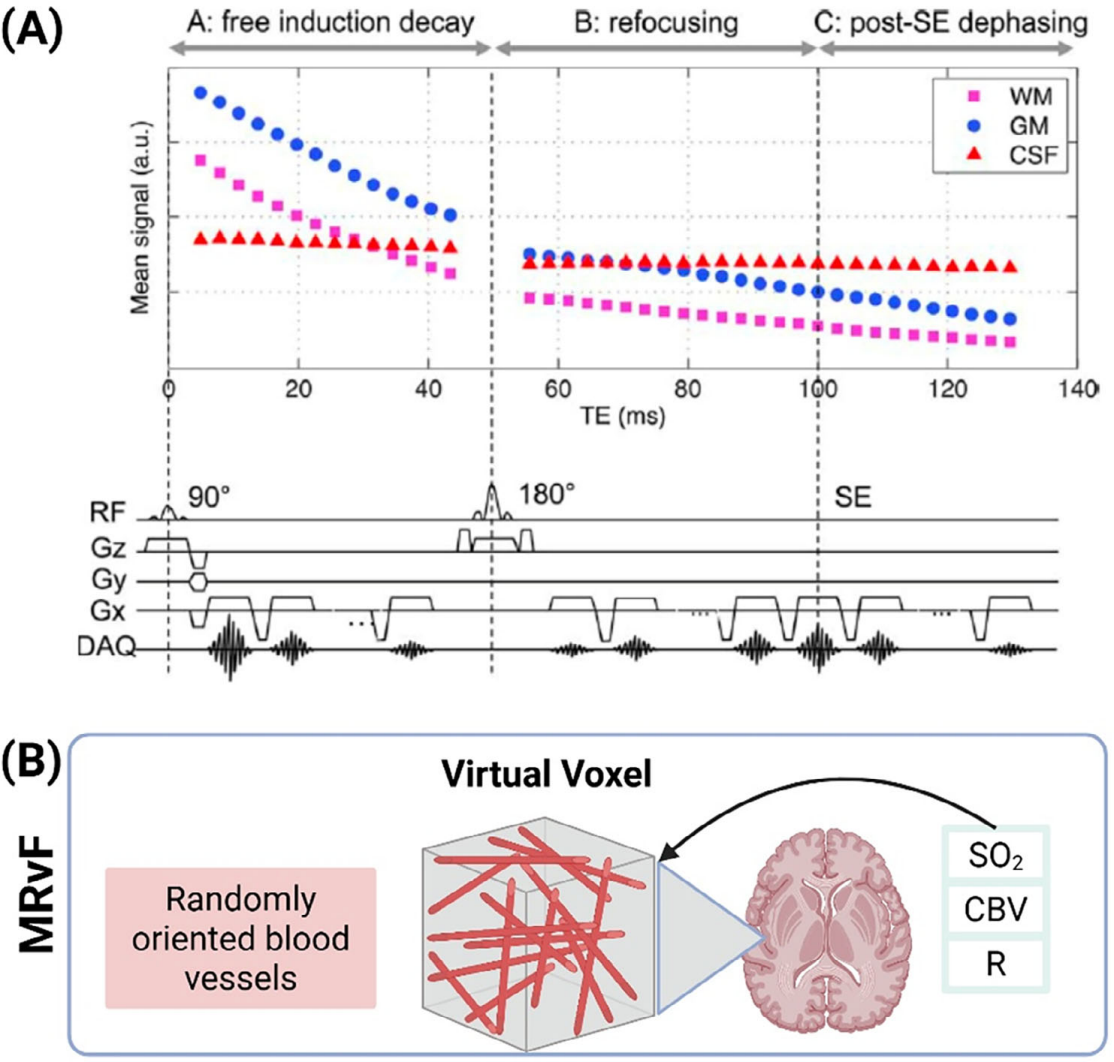
MRvF acquisition and modeling framework (adapted from Ni et al. [19], *Magn Reson Med* 2015). (A) Schematic of the gradient‐echo sampling of free‐induction decay and spin‐echo (GESFIDE) sequence. The pulse train (RF and gradient timing) captures FID, SE, and post‐SE signal components sensitive to susceptibility‐induced dephasing and microvascular architecture. Representative signal evolutions for white matter (WM), gray matter (GM), and cerebrospinal fluid (CSF) demonstrate tissue‐specific contrast behavior across echo times. (B) Biophysical model used in MRvF: A virtual voxel containing randomly oriented cylindrical vessels simulates the MR signal as a function of oxygen saturation (SO_2_), cerebral blood volume (CBV), and vessel radius (R). Matching the in vivo signal trajectory to this simulated dictionary enables quantitative mapping of vascular physiology.

As illustrated in Figure 3A, MRvF commonly employs a multi‐echo GESFIDE sequence, which samples free‐induction decay, SE, and post‐SE dephasing signals across multiple echo times to capture both extravascular and intravascular susceptibility effects. The RF and gradient timing structure provides separation of relaxation and dephasing contributions, making the signal evolution sensitive to microvascular geometry and oxygenation. This signal dependence on vessel orientation and deoxyhemoglobin concentration provides rich temporal features that are subsequently matched against simulated fingerprints to estimate vascular parameters. In the modeling step (Figure 3B), a biophysical Monte Carlo simulation is performed within a virtual voxel containing randomly oriented cylindrical vessels that mimic the brain's microvascular architecture. The simulated signal evolution varies with input parameters, SO_2_, CBV, and vessel radius R forming a comprehensive dictionary for fingerprint matching. By comparing acquired in vivo signal trajectories with this simulated dictionary, MRvF shows voxel‐wise estimation of physiological maps reflecting cerebral oxygenation, vascular density, and microvascular caliber.

Beyond the GESFIDE framework, Coudert et al. introduced a balanced steady‐state free precession (bSSFP)‐based MRvF variant that capitalizes on bSSFP's outstanding sensitivity to intra‐voxel frequency shifts, which arise from microvascular susceptibility gradients and its exceptionally high SNR efficiency, to directly encode microvascular features such as CBV, vessel radius (R) and oxygenation into the MRF signal [44].

### 3.2 Dictionary Simulation

Signal simulation in MRvF involves seeding a virtual voxel with randomly oriented micro‐vessels to mimic the deoxyhemoglobin‐related susceptibility gradients and diffusion‐driven phase dispersion that govern the gradient‐echo (GRE) and spin echo (SE) signal (Figure 3A). Simulations typically model the effects of water proton diffusion in magnetic field gradients within a virtual voxel containing randomly oriented blood vessels. Because both intravascular and extravascular compartments are followed, the resulting fingerprints are exquisitely sensitive to CBV, R, and oxygen‐dependent susceptibility shifts to allow quantitative maps of SO_2_ that conventional BOLD cannot supply. The simulated parameters typically cover a wide range corresponding to both healthy and pathological conditions.

To emulate more realistic cerebrovasculature, more advanced simulations incorporate detailed vascular geometries derived from high‐resolution angiograms demonstrated by Pouliot et al. [27]. Such models can account for intricate features like vessel tortuosity, hierarchical branching patterns, and regional heterogeneity in vascular architecture to offer a more reliable representation of the cerebral complex vasculature. By using Monte Carlo methods to model the effects of local magnetic field inhomogeneity and superparamagnetic iron oxide nanoparticles (SPION) if contrast is used, this approach allows for a more accurate simulation of vascular‐related parameters and improves the robustness of MRvF in preclinical and clinical settings. Recent studies by Delphin et al. demonstrated that dictionaries built from realistic 3D microvascular geometries (e.g., segmented mouse brain) produce parameter estimates that align more closely with both histology and optical‐fiber oxygen measurements in tumors, compared to cylinder models [31, 45].

However, MRvF faces challenges in accurately modeling the complexity of brain microvasculature, including vessel orientation, size distribution, and oxygenation [46]. Its conventional analytical framework relies on the classical qBOLD model, which assumes blood vessels are randomly oriented infinite cylinders of the same basis used in the foundational Yablonskiy et al. study [47, 48]. While this cylindrical approximation simplifies the mathematics, it imposes limitations that cannot fully capture heterogeneous vessel size distributions, non‐random orientation patterns, or pathological alterations. Recent advances have included the use of realistic vascular geometries and Monte Carlo simulations to better capture the vascular heterogeneity of pathological conditions, such as atherosclerosis and tumors [27]. Machine learning approaches have also been introduced to improve the efficiency and accuracy of parameter estimation, particularly for small vessels and complex vascular networks [49, 50]. Figure 2B in the review illustrates how MRvF uses a virtual voxel approach with randomly oriented blood vessels consistent with qBOLD theory to model microvascular architecture [51].

Further dictionary generation is introduced by Coudert et al., who utilized a bSSFP‐based MRvF approach to leverage the sensitivity to intra‐voxel frequency shifts and its high SNR efficiency to directly encode microvascular features into the MRF signal. Their methodology involved a two‐step dictionary generation process. An initial base dictionary covered fundamental MR parameters (T_1_, T_2_, B_1_, and off‐resonance frequency shift *δ*_*f*_). This foundation was subsequently expanded into a comprehensive vascular dictionary that incorporated both Lorentzian and 3D microvascular voxel‐based models of intra‐voxel frequency distributions. The bSSFP‐based MRvF approach capitalizes on higher intrinsic SNR and enhances sensitivity to intra‐voxel frequency variations, making it effective for encoding subtle microvascular features [52]. GESFIDE is a fixed multi‐echo sequence, which offers more direct and stable separation of T_2_ and T_2_* components without the susceptibility‐related banding artifacts seen in bSSFP with lower overall SNR and reduced susceptibility contrast.

### 3.3 In Vivo Mapping of Cerebral Vascular Parameters

Initial demonstrations by Christen et al. utilized a 2D GESFIDE sequence in conjunction with the contrast agent ferumoxytol. They acquired data before and 2 min after contrast administration and defined the vascular fingerprint as the ratio of pre‐ to post‐contrast signals, effectively canceling out confounding factors such as static B_0_/B_1_ inhomogeneities and T_2_ relaxation effects. This approach enabled robust voxel‐wise mapping of CBV, vessel radius, and SO_2_ in healthy human subjects to establish the fundamental feasibility of MRvF in vivo and yield parameter values consistent with conventional imaging benchmarks. In vivo MRvF mapping proceeds by comparing each voxel's normalized signal evolution (or “fingerprint”) acquired via multi‐echo GESFIDE to a Monte Carlo derived dictionary spanning combinations of CBV, R, and SO_2_. Each voxel is assigned the parameters corresponding to the dictionary entry with the highest inner‐product match, completing the vascular parameter map.

A significant trajectory in MRvF development has been the pursuit of contrast‐free methods for broadening its applicability and enhancing patient safety. Wheeler et al. utilized a contrast‐free SAGE sequence combined with an iterative matching algorithm in five echoes to achieve a temporal resolution of less than 5 s per vascular map for dynamic measurements such as cerebrovascular reactivity (CVR) during hypercapnia [28]. Their CVR maps revealed distinct regional differences, with higher CBV and SO_2_ reactivity observed in cortical gray matter compared to white matter, consistent with known physiological patterns. This work is important as it transforms MRvF from a predominantly static microstructural mapping tool into a dynamic technique capable of assessing the functional responses of the microvasculature to physiological challenges, without the need for exogenous contrast. Similarly, Coudert et al. introduced a contrast‐free MRvF method employing a bSSFP sequence [44]. This approach allowed for the quantification of CBV, R, and standard relaxometry parameters (T_1_, T_2_, T_2_*), demonstrating improved differentiation of CBV and R in gray versus white matter that aligned well with established literature values.

Recent efforts have extended MRvF by integrating deep learning approaches to accelerate and refine the parameter estimation process. Lin et al. introduced a neural network trained on simulated GESFIDE‐based dictionaries, successfully estimating CBV, vessel radius, and SO_2_ with higher accuracy compared to traditional dictionary matching [53]. More recently, Barrier et al. introduced the MARVEL framework by employing bidirectional Long Short‐Term Memory (LSTM) networks trained on realistic 3D vascular simulations derived from microscopy data [50]. This deep learning model simultaneously recovered relaxometry (T_1_, T_2_), B_0_/B_1_ inhomogeneity, and microvascular parameters, offering rapid, high‐quality maps without the need for contrast agents.

### 3.4 Reproducibility

Validating microvascular imaging techniques that obtain true “ground truth” for parameters like in vivo vessel radius or SO_2_ at a microscopic level is often invasive or impossible in human subjects. Therefore, validation frequently relies on comparisons with other indirect imaging methods or on consistency with known physiological principles. In preclinical cerebral hypoxia/ischemia and glioma models, qBOLD frameworks combining with T_2_, T_2_*, and CBV measurements have demonstrated strong linear correlations (R^2^ = 0.9) between MRI‐derived SO_2_ and invasive co‐oximetry across graded levels of inspired oxygen, with MRI‐demarcated hypoxic regions aligning spatially with histologic hypoxia markers [54]. Although human ground‐truth measurements at the micro‐vessel level remain impractical, alternative approaches such as calibrated BOLD/OEF [55] and T_2_‐relaxation under spin‐tagging (TRUST) [56] MRI have been evaluated against PET or controlled venous oximetry, reporting reproducibility (e.g., intraclass correlation = 0.90) and error margins consistent with expected physiologic variations [57].

## 4 Vascular Fluid Dynamics (VFD)‐MRF

### 4.1 Physiological Parameters and Acquisition

MRF‐VFD refers to specialized MRF acquisitions and analytic frameworks devised to quantitatively characterize the movement of fluids through tracer molecules and plasma across vascular and tissue compartments. This includes the assessment of BBB permeability and the characterization of DCE MRI parameters, offering insights into the integrity and function of the neurovascular unit.

**FIGURE 4:**
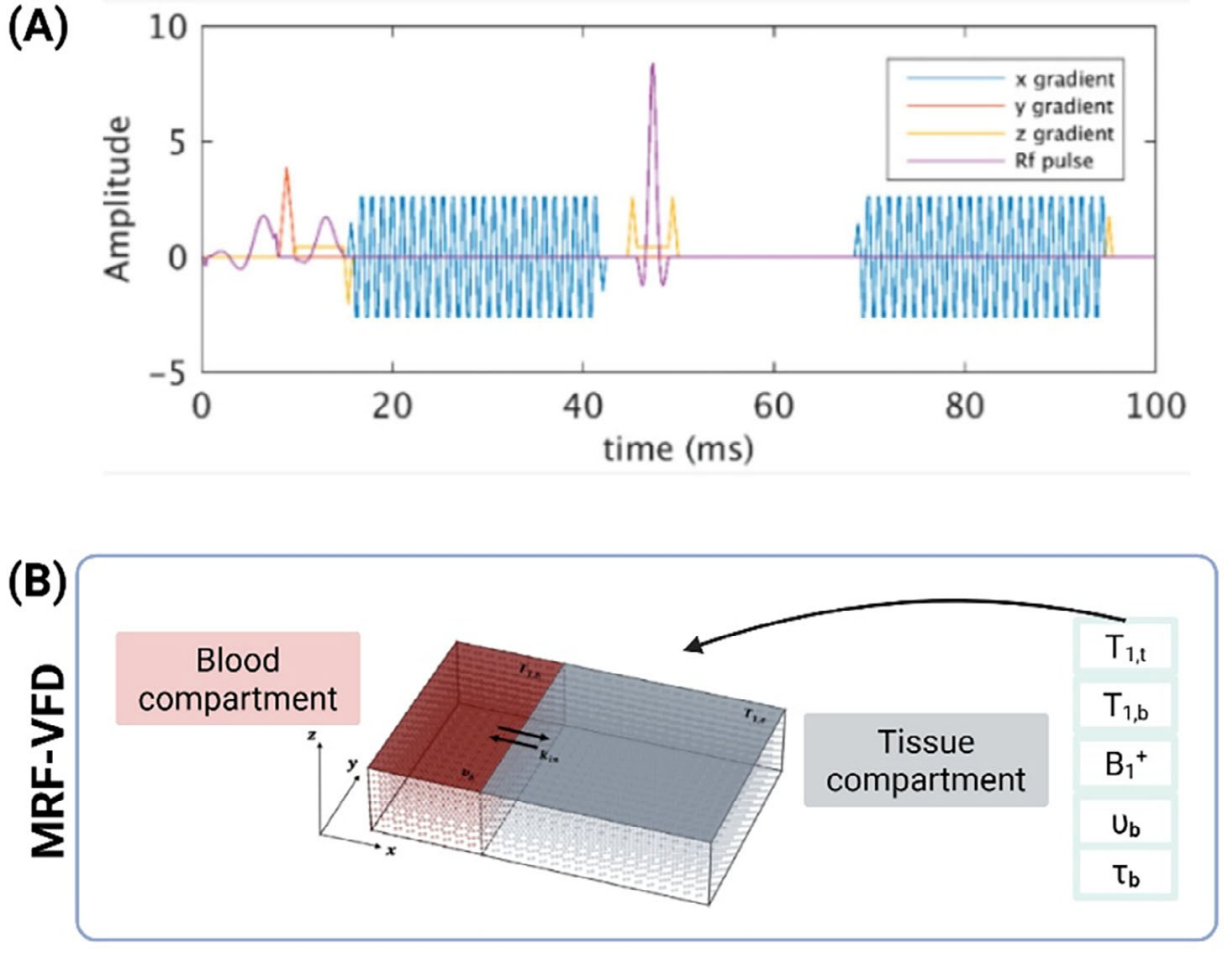
Acquisition and modeling framework for MRF‐VFD. (A) Pulse‐sequence schematic adapted from Venugopal et al. [34]: A gradient‐echo–based MRF schedule alternating RF pulses and *x*/*y*/*z* gradient lobes to encode sensitivity to fluid exchange and microvascular dynamics. (B) Biophysical two‐compartment model adapted from Thomson et al. [35]: A blood compartment (red) and a tissue compartment (gray) coupled by water‐exchange parameters including intravascular residence time (τ_b_), transfer constant (K_in_), compartmental relaxation times (T_1_,_t_, T_1_,_(b)_), blood velocity (υ_b_), and transmit field (B_1_^+^). These parameters are incorporated into the simulation dictionary for quantitative matching of in vivo fingerprints.

As shown in Figure 4A (adapted from Venugopal et al. [34]), MRF‐VFD employs a gradient‐echo–based pulse sequence that alternates RF pulses and *x*/*y*/*z* gradient lobes to encode dynamic susceptibility and relaxation effects associated with intravascular and extravascular fluid exchange. The temporal variation of the applied gradients and RF pulses modulates sensitivity to inflow, diffusion, and leakage phenomena, forming a unique signal evolution for each voxel that reflects underlying BBB transport processes. The biophysical model (Figure 4B, adapted from Thomson et al. [35]) divides the voxel into blood and tissue compartments coupled through exchange parameters such as the intravascular water residence time (τ_b_), blood‐to‐tissue transfer constant (K_in_), and compartmental relaxation times (T_1_,_t_, T_1_,_b_). The model also includes transmit‐field inhomogeneity (B_1_^+^) and blood velocity (υ_b_) terms to account for flow‐related signal modulation. These parameters jointly determine the simulated signal dictionary used for quantitative estimates of BBB transport properties without relying on contrast leakage assumptions.

A central challenge in MRF‐VFD is the accurate modeling of complex tracer kinetics, particularly when assessing BBB integrity [36, 58]. For methods employing exogenous contrast agents, such as in DCE‐MRI studies, the passage of the agent and its potential leakage across a compromised BBB significantly influence the MR signal. Contrast agent extravasation can alter local T_1_ and T_2_* relaxation times to complicate the quantification of perfusion parameters like rCBV and permeability measures like K_trans_ [59].

In DCE‐MRI based MRF‐VFD, the administration of a preload contrast dose intended to saturate rapid leakage effects before the main dynamic acquisition can itself influence parameter estimation. Van Dorth et al. investigated this aspect and found that omitting a preload dose improved the estimation of permeability [36]. This observation was attributed to the enhanced sensitivity of the GRE and SE signals to subtle leakage effects during the initial passage of the first bolus. However, the same study reported that preload omission compromised the accuracy of rCBV estimation, especially in regions with heterogeneous vascular architecture, where a second bolus (effectively experiencing a preload from the first) provided better rCBV resolution. These findings highlight that choices in acquisition strategy, such as the use of a preload, involve inherent trade‐offs and can differentially affect the accuracy of various physiological parameters.

For non‐contrast MRF‐VFD methods that aim to measure BBB water permeability directly (e.g., by quantifying intravascular water residence time, τ_b_, as in the work of Thomson et al. [35]), the primary challenge lies in detecting the extremely subtle MR signal changes associated with water molecules exchanging across the BBB. These subtle modulations must be reliably distinguished from the background signals arising from other physiological processes and inherent MR relaxation effects. Such methods necessitate highly sensitive acquisition sequences and robust biophysical models capable of isolating these minute signal variations. The development of non‐contrast methods for assessing BBB integrity is a crucial frontier, as it eliminates exposure to gadolinium‐based contrast agents and provides safer, repeatable monitoring of subtle BBB alterations over time without the associated safety concerns or contraindications [60].

### 4.2 Dictionary Simulation

Accurate rCBV estimation in MR‐VFD presents a circular challenge: correcting for leakage depends on perfusion assumptions, while perfusion modeling itself can be skewed by leakage effects. This interdependence demonstrates the importance of biophysical models that can cleanly separate intravascular and extravascular/extracellular contributions to ensure reliable assessment of BBB permeability and water exchange. Dictionary simulation in MRF‐VFD is designed to model the dynamic processes of fluid movement and exchange within the cerebral vasculature (Figure 4B). For techniques based on DCE‐MRI, the dictionaries are constructed to simulate the MR signal evolution throughout the passage of an exogenous contrast agent bolus. This often involves intricate sequence modeling with varying parameters, such as the FA, which is coupled to a time‐varying blood T_1_ curve that reflects the changing concentration of the contrast agent in the blood. Physiological parameters incorporated into these dictionary entries typically include R, rCBV, and permeability metrics such as K_trans_ or k. The role of a dictionary is crucial in disambiguating various overlapping kinetic processes such as blood flow delivering the contrast agent, the agent filling the intravascular space, its potential leakage into the extravascular space, and subsequent washout. The dynamically varying acquisition parameters aim to sensitize the signal differently to these processes at various time points, and the dictionary must accurately model this complex interplay.

To capture rapid contrast dynamics effectively, high temporal resolution in both acquisition and simulation is essential. Gu et al. developed dictionaries for a fast MRF approach (utilizing bSSFP and fast imaging with steady‐state precession (FISP) sequences with spiral trajectories) specifically for DCE‐MRI studies in mice [33]. Their method provided simultaneous T_1_ and T_2_ mapping with a high temporal resolution of approximately 2 min, facilitating the assessment of BBB integrity. This dictionary includes T_1_, T_2_, and off‐resonance frequency parameters. T_1_ is the most sensitive marker of BBB leakage, as gadolinium accumulation in the extravascular extracellular space can shorten T_1_ in proportion to contrast concentration changes. In contrast, T_2_ changes tend to be more subtle, and off‐resonance primarily reflects magnetic field inhomogeneity, offering no direct information about permeability. For non‐contrast MRF‐VFD methods focusing on BBB water exchange, such as the technique developed by Thomson et al., dictionary simulation is optimized to detect very subtle signal changes [35]. Their approach used an RF‐spoiled gradient echo MRF sequence to quantify CBV and the intravascular water residence time (τ_b_), which is a parameter related to BBB water permeability. The dictionary simulation was optimized using Bloch equation calculations for multiband RF pulses, with the primary goal of maximizing sensitivity to CBV and τ_b_ through strategic variations in FA and TR. The dictionary entries for this method included parameters for blood T_1_, tissue T_1_, CBV, τ_b_, and the relative transmit field (B_1_^+^). Such simulations must be based on highly precise biophysical models capable of predicting how minute shifts in water distribution will alter the MRF fingerprint, distinguishing these effects from simple T_1_ relaxation.

MRF‐VFD dictionaries must also be adaptable to various contrast administration protocols, including multi‐bolus injections and strategies involving preload doses. Van Dorth et al. simulated signal evolution for a two‐bolus DSC‐MRI acquisition (with and without the effects of a preload) using a hybrid GRE/SE sequence. Their dictionary, containing 5000 entries, modeled both GRE and SE signal dynamics for unique combinations of permeability, R, and rCBV, allowing them to systematically assess how preload conditions affect parameter estimation.

### 4.3 In Vivo Mapping of Cerebral Vascular Parameters

Recent study by MacAskill et al. assessed tumor vascular perfusion and permeability in vivo [32]. They performed preclinical studies in 25 mice bearing orthotopic 4 T_1_ breast tumors, generating voxel‐wise gadolinium concentration curves directly from T_1_ evolution and estimating perfusion metrics via the linear reference‐region model. Compared with conventional DCE‐MRI, DCE‐MRF achieved a two‐ to three‐fold reduction in temporal variability of T_1_ measurements and produced consistent pharmacokinetic estimates of relative volume transfer constant (RK^trans^) and blood perfusion parameter (k_ep_). In vivo maps revealed markedly higher RK^trans^ (0.54 ± 0.11 a.u.) and k_ep_ (0.083 ± 0.012 min^−1^) in the tumor rim compared with the core (0.11 ± 0.05 a.u. and 0.058 ± 0.009 min^−1^; *p* < 0.001), consistent with increased vascular permeability and perfusion at the proliferative tumor margin. The study also demonstrated the feasibility of a 7.5 s human‐scanner DCE‐MRF prototype in a rabbit kidney, indicating potential clinical translatability for future oncologic and cerebral perfusion applications.

**FIGURE 5:**
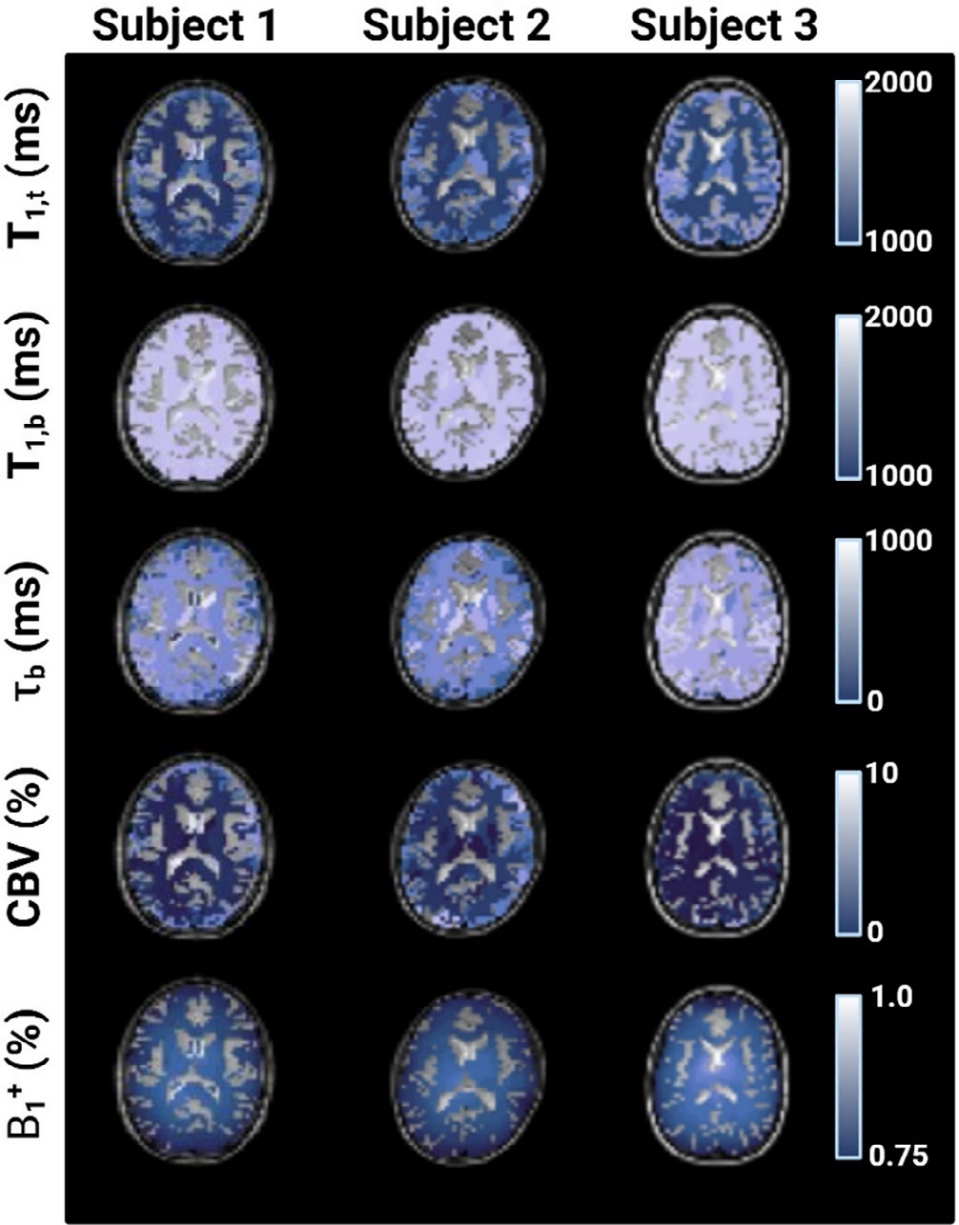
Quantitative parameter maps of intravascular T_1_ (T_1,b_), extravascular T_1_ (T_1,t_), intravascular water residence time (τ_b_), CBV, and relative transmit field (B_1_^+^) generated using the non‐contrast MRF method in three healthy volunteers. Adapted from Thomson et al. [35], *Magn. Reson. Med*. 2024.

Gu et al. demonstrated the utility of a fast MRF method for simultaneous T_1_ and T_2_ mapping to achieve high‐temporal resolution imaging of contrast agent dynamics and BBB integrity within DCE‐MRI studies in mice. After acquiring in vivo data, the signal time course of each voxel was compared against a precomputed dictionary containing signal evolutions simulated across combinations of parameters, that is, T_1_, T_2_, off‐resonance. Matching was performed using a normalized inner product (dot‐product) criterion. Similarly, Thomson et al. utilized inner product matching for their novel non‐contrast MRF sequence to quantify CBV and τ_b_ in healthy volunteers (Figure 5). The findings were consistent with known physiology that gray matter has higher CBV compared to white matter with a non‐contrast approach. Interestingly, no significant regional differences were found for τ_b_ or blood T_1_ in their healthy cohort, suggesting relatively uniform blood oxygenation and baseline water exchange rates across these regions.

The practical implications of acquisition strategy choices in MRF‐VFD have also been explored in vivo. Van Dorth et al. employed their two‐bolus DSC‐MRI MRF‐VFD technique in glioma patients to investigate the impact of omitting a preload contrast dose. Their findings indicated that while permeability maps showed slightly elevated values in tumor regions during the second bolus (where the first bolus effectively acted as a preload), rCBV maps derived from this second bolus exhibited greater contrast. This observation aligned with their simulation predictions, which suggested that preload omission, while potentially beneficial for permeability estimation, could compromise rCBV accuracy in tissues with heterogeneous vascular architecture. Such studies provide valuable, practical insights for optimizing MRF‐VFD protocols depending on the specific clinical question or the primary physiological parameter of interest.

### 4.4 Reproducibility

Venugopal et al. validated their MRF‐VFD (hybrid DSC) derived rCBV and R maps against conventional VSI in their study of glioma patients. They reported that the MRF‐VFD technique exhibited high consistency with VSI, but importantly, it also demonstrated improved robustness to noise and enhanced spatial resolution. While the overall agreement was high, they acknowledged the potential confounding effect of contrast agent leakage on rCBV measurements in some cases, as well as the known limitations of VSI, particularly in the accurate sizing of larger vessels. Validating dynamic parameters like K_trans_ or τ_b_ is challenging due to their sensitivity to transient physiological states and complex tissue–contrast interactions. These factors introduce variability that complicates reproducibility, underscoring the need to refine acquisition strategies and biophysical models to improve the reliability and translational potential of MRF‐VFD.

## 5 Clinical Applications of Physiological MRF

The translation of physiological MRF has sought to demonstrate the power of quantitative, multi‐parametric imaging of underlying pathophysiology across a spectrum of neurological disorders. In cerebrovascular diseases, these techniques can precisely map the extent of hemodynamic compromise and reveal compensatory mechanisms that are invisible to conventional imaging. In neuro‐oncology, they offer a window into the tumor microenvironment, quantifying hypoxia and vascular remodeling, which are critical for grading tumors and predicting treatment response. The clinical performance and utility of each modality in physiological MRF depend directly on the technical factors we have reviewed. The achievable spatial and temporal resolution, signal‐to‐noise constraints, motion sensitivity, calibration or normalization strategies, and modeling assumptions all affect not only measurement fidelity but also robustness in patient populations. Thus, the following sections highlight representative studies, summarized in Table 3, that showcase the clinical utility of physiological MRF to characterize disease pathophysiology.

**TABLE 3:** Summary of key findings from studies employing physiological MRF techniques for cerebral physiology and tumor analysis.

	Disease type	Sample size	Technique	Physiological parameters	Key findings
Pouliot et al. [27]	Atherosclerosis	10 ATX and 10 healthy WT mice	MRvF	SO_2_, CBV, and R	ATX mice showed increased R (9.2 ± 0.3 μm vs. 7.4 ± 0.2 μm in WT), elevated CBV (8.6% ± 0.4% vs. 6.9% ± 0.3%), and reduced SO_2_ (48% ± 3% vs. 62% ± 2%)
Zhang et al. [38]	Moyamoya disease	7 healthy subjects and 3 patients	Deep‐learning‐based MRF‐ASL	B_1_^+^, T_1_, CBF, tBAT, pass‐through arterial BAT, pass‐through CBV, and pass‐through blood travel time	Reduced computation time (< 0.5 ms/voxel vs. >4 s for dictionary matching); accurately localized regions of diminished CBF and prolonged BAT; aligned with occluded arteries in MR angiography
Su et al. [39]	Moyamoya disease	21 patients and 16 controls	MRF‐ASL	BAT, CBF, and T_1_	BAT in stenotic ICA territories was prolonged (1900 ± 300 ms vs. 1100 ± 200 ms in controls); CBF preserved in ICA regions but elevated in PCA territories (50 ± 7 mL/100 g/min vs. 35 ± 6 mL/100 g/min in controls)
Fan et al. [61]	Ischemic Stroke	34 patients	MRF‐ASL	CBF_1_‐compartment, CBF_2_‐compartment, BAT, and T_1_	CBF in stroke lesions was 15.1 ± 4.9 mL/100 g/min vs. 28.6 ± 5.3 mL/100 g/min in normal tissue; BAT prolonged in lesions (1500 ± 300 ms vs. 900 ± 200 ms). MRF‐ASL outperformed pCASL in stroke lesion classification and severity assessment
Lemasson et al. [62]	Brain tumors and stroke	115 rats (rat models: 9L, C6, F98)	MRvF	SO_2_, CBV, and R	MRvF effectively distinguishes pathological from healthy tissue; lower SO_2_ and larger vessel radii in 9L gliomas compared to qBOLD
Venugopal et al. [34]	Gliomas	6 patients	MRvF	rCBV and R	MRvF shows elevated rCBV and heterogeneous vessel radii in gliomas aligning well with DSC imaging

Abbreviations: ATX, atherosclerotic; CBF, cerebral blood flow; CBV, cerebral blood volume; ICA, internal carotid artery; PCA, posterior carotid artery; pCASL, pseudo‐continuous arterial spin labeling; R, vessel radius; SO_2_, oxygen saturation; tBAT, tissue bolus arrival time; WT, wild type.

### 5.1 Cerebrovascular Diseases

Physiological MRF has emerged as a powerful tool for studying cerebrovascular diseases for detailed insights into microvascular architecture and function. Its ability to simultaneously quantify multiple vascular parameters, such as CBV, SO_2_, and R, positions it as a natural choice for investigating conditions characterized by vascular remodeling and dysfunction.

Pouliot et al. explored MRvF in ATX and healthy WT mice, focusing on microvascular characteristics in the brain's cortical and subcortical regions [27]. The study utilized realistic angiograms for dictionary‐based parameter extraction, revealing significant physiological differences. Aged ATX mice exhibited a mean vessel radius of 9.2 ± 0.3 μm, significantly larger than 7.4 ± 0.2 μm in WT mice, reflecting vascular remodeling. Additionally, SO_2_ was markedly reduced in ATX mice at 48% ± 3% compared to 62% ± 2% in WT. CBV was also elevated in ATX mice at 8.6% ± 0.4%, versus 6.9% ± 0.3% in WT. These findings showed the capability of detecting atherosclerosis‐driven microvascular changes linked to cerebrovascular dysfunction.

In Zhang et al. (DeepMARS), MRF‐ASL parametric maps reconstructed in seven healthy subjects and three Moyamoya patients showed increased BAT and decreased CBF precisely co‐localized with occluded arteries on TOF angiography [38]. DeepMARS achieved ultra‐fast computation (< 0.5 ms/voxel vs. > 4 s) and higher ICC and R^2^ than conventional template matching, with a strong correlation to Look‐Locker PASL estimates of BAT and CBF (R^2^ > 0.90) [63]. In Su et al. (21 Moyamoya patients, 16 controls) [39], BAT within stenotic ICA/ACA/MCA regions was significantly increased (≈1470 ± 264 ms vs. 964 ± 121 ms in controls, *p* < 0.001), yet basal CBF remained comparable (∼34 ± 7 mL/100 g/min) in those same regions, while posterior circulation CBF was elevated (∼40 ± 8 vs. 31 ± 6, *p* < 0.01), suggesting compensatory flow. MRF‐ASL BAT correlated significantly with MRA stenosis grading (Spearman *r* = 0.52, *p* < 0.001), whereas CBF showed no such relationship. The two studies were broadly consistent in identifying delayed BAT in stenotic anterior circulation in Moyamoya. The divergence in CBF findings likely reflects differences in cohort size, disease severity, and collateral adaptation that Zhang's small cohort showed local CBF deficits, while Su's larger cohort captured flow preservation in the anterior region and augmentation via posterior collateral pathways.

**FIGURE 6:**
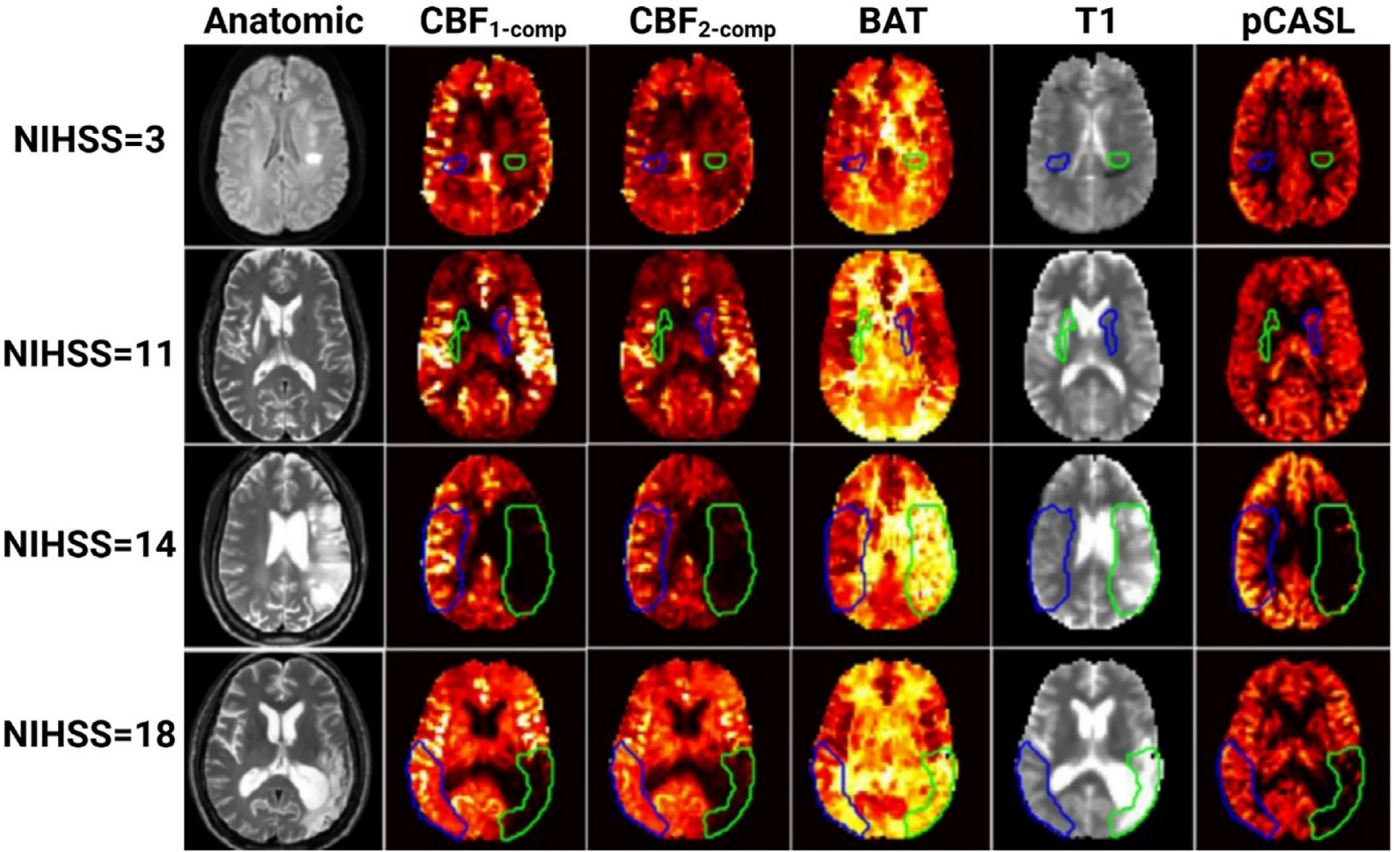
Representative images of MRF‐ASL, traditional pCASL, and anatomical MRI in four stroke patients with NIHSS scores of 3, 11, 14, and 18. Maps include CBF_1_‐comp, CBF_2_‐comp, bolus arrival time (BAT), and T_1_ from MRF‐ASL, with T_1_ representing quantitative measurements (darker signals = longer T_1_). Anatomical images (DWI or T_2_‐weighted) delineate lesions (green) and normal tissue (blue), highlighting perfusion and tissue differences across stroke severities. Adapted from Fan et al. [61], *Stroke*, 2022.

Fan et al. investigated MRF‐ASL for ischemic stroke imaging [61]. Using a cohort of 34 stroke patients, the study compared stroke lesions, perilesional tissues, and contralateral normal regions (Figure 6). Quantitative metrics revealed lower CBF in stroke lesions, with single‐compartment CBF_1_‐compartment at 15.1 ± 4.9 mL/100 g/min compared to 28.6 ± 5.3 mL/100 g/min in normal tissue, and extended BAT of 1500 ± 300 ms in lesions versus 900 ± 200 ms in normal regions. Tissue T_1_ values in lesions were prolonged (1200 ± 100 ms vs. 1050 ± 70 ms). These parameters correlated with neurological severity scores (National Institutes of Health Stroke Scale: NIHSS, and modified Rankin Scale: mRS) and showed superior stroke lesion classification performance compared to traditional pCASL.

### 5.2 Tumor Characterization and Monitoring

The application of MRF to quantify maps of T_1_, T_2_, and proton density in brain tumor imaging has enabled detailed characterization of tumor microenvironment and heterogeneity. These unique relaxation signatures from MRF can distinguish between tumor types, such as glioblastomas, low‐grade gliomas, and metastases [64]. Understanding the microvascular environment of tumors, comprising various cell types and extracellular components, is clinically significant in cancer progression and therapeutic resistance [65].

Lemasson et al. explored MRvF in preclinical rat models of brain tumors (9L, C6, and F98) and stroke [62]. By simultaneously mapping SO_2_, CBV, and R, MRvF demonstrated its ability to differentiate between healthy and pathological brain tissues. Physiologically, 9L is highly immunogenic and prone to extensive neovascular changes, including large vessel formation and altered microvasculature. Conversely, C6 tumors are less vascular but moderately hypoxic, and exhibit intermediate oxygenation and angiogenic remodeling. F98 tumors are weakly immunogenic, diffusely invasive, and show relatively higher perfusion and oxygenation compared to 9L. The study revealed that the 9L glioma exhibited significantly lower SO_2_ compared to both C6 and F98, indicating a more hypoxic environment. For example, MRvF quantified SO_2_ in the 9L glioma model at approximately 30%, while C6 and F98 gliomas showed higher values, around 45%–50%, reflecting differences in vascular oxygenation and metabolic demand. These findings were different from the results obtained using non‐MRF qBOLD imaging, which overestimated SO_2_ in the 9L glioma model at 40%–50%.

Venugopal et al. applied a MRvF using a hybrid gradient–spin echo DSC‐MRI approach in six glioma patients [34]. The study quantified rCBV and R and compared these results with the conventional VSI method. MRvF showed high consistency with VSI but demonstrated improved robustness to noise and enhanced spatial resolution. Vessel radius values ranged from 5 to 150 μm, and rCBV values varied between 0.5% and 10%, which were significantly elevated compared to healthy tissues, reflecting the abnormal vascular proliferation and heterogeneity characteristic of gliomas [66]. These findings provide non‐invasive markers for glioma grading and monitoring of treatment response through quantitative vascular metrics [67, 68, 69].

**FIGURE 7:**
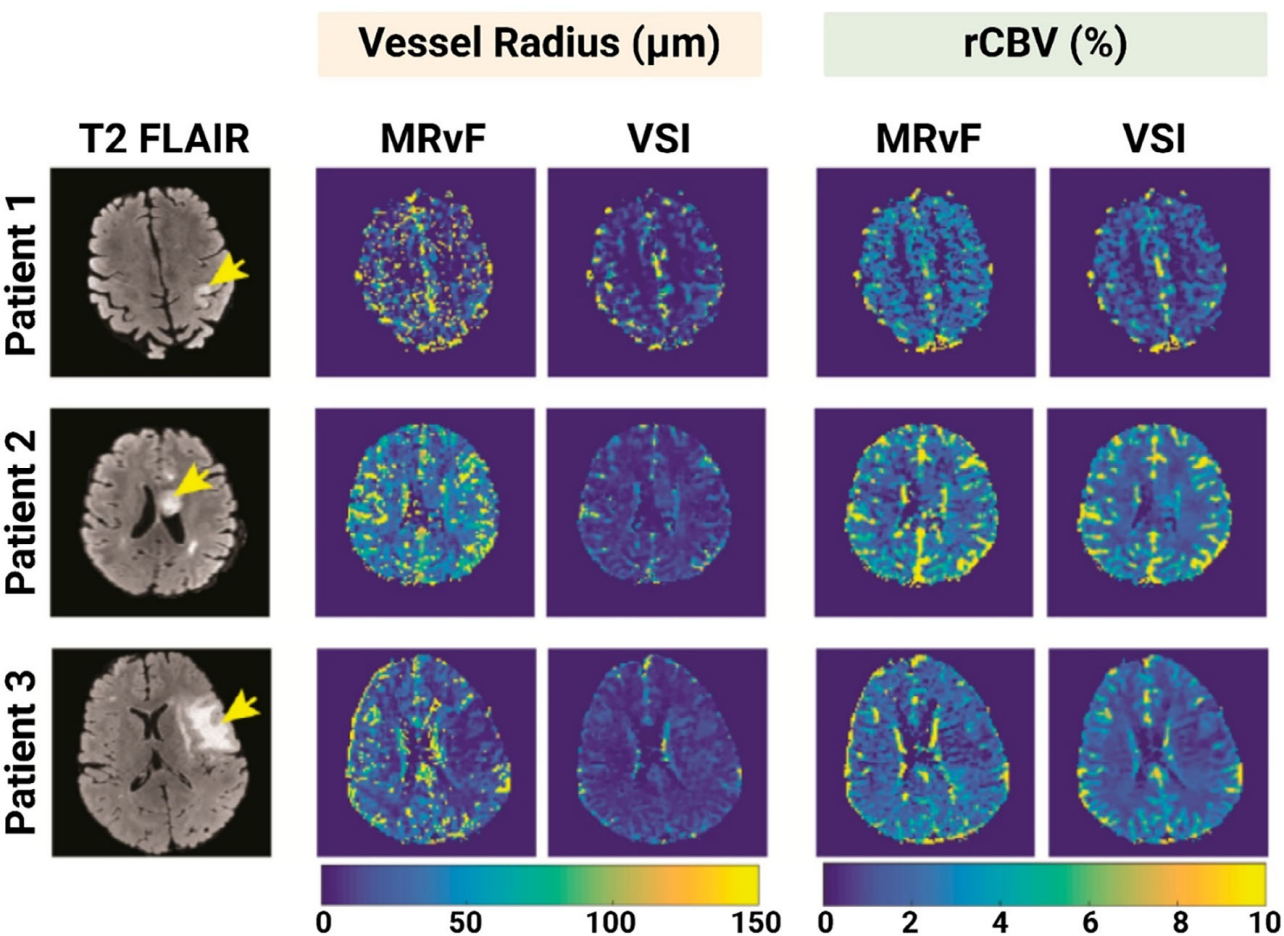
Comparison of R (μm) and rCBV (%) maps derived from MRvF and the Vessel Size Imaging (VSI) method for three patients with gliomas. T_2_ FLAIR images (left column) highlight tumor regions (yellow arrows). The MRvF‐derived maps (middle columns) show vessel radius and rCBV values alongside those derived from VSI (right columns). Adapted from Venugopal et al. [34], *Cancers*, 2023.

Figure 7 compares MRvF and conventional VSI in glioma patients, showing maps of R (μm) and rCBV (%). Both methods generated qualitatively similar patterns in which tumor regions exhibit higher rCBV and slightly larger vessel radius compared to normal tissue, consistent across three patients in one case, MRvF produced higher rCBV than VSI, possibly reflecting leakage effects in high‐grade tumors. Independent validation in human gliomas confirmed VSI MRI estimates of mean vessel radius (˜13.7 μm) closely matched histology (˜12.6 μm); rCBV correlated with vessel density (*r* = 0.42, *p* = 0.03) while VSI correlated with vessel size (*r* = 0.49, *p* = 0.01) [70, 71]. Overall, Venugopal et al. showed the high consistency of estimated parametric maps in the two techniques. However, additional comparisons are needed especially in larger vessel sizes [72] (where VSI shows underestimation) that occur in tumor tissues with increased blood supply to support their rapid growth [73].

## 6 Technical Considerations and Future Advancements in Physiological MRF Studies

### 6.1 Field Inhomogeneity and SNR Trade‐Offs in Physiological MRF

Robust physiological MRF hinges on acquisition sequences that minimize sensitivity to magnetic field inhomogeneity (both B_0_ and B_1_^+^), which otherwise introduces parameter bias and artifacts. The impact of field inhomogeneity has directly influenced sequence design and methodological choices in physiological MRF. The most rigorous approach is to acquire subject‐specific maps of static (B_0_) and transmit RF (B_1_^+^) fields and incorporate them into the dictionary simulation. However, this strategy requires additional scans to increase total acquisition time and experimental complexity. An alternative strategy is to employ pulse sequences that are inherently less sensitive to field variations. For example, while early MRF protocols based on bSSFP offered high SNR, they were highly susceptible to B_0_ inhomogeneity [18]. Consequently, many physiological MRF applications favor spoiled GRE‐based sequences, such as FISP [74] or FLASH [75]. These variants provide substantially greater robustness to B_0_ variations, making them a more practical choice for in vivo applications. On the reconstruction front, deep‐learning–based correction frameworks offer scan‐specific field calibration without additional acquisitions. For example, Kang et al. introduced an “Only‐Train‐Once” recurrent neural network capable of ingesting measured B_0_ and B_1_ maps at runtime to automatically correct for inhomogeneities across various MRF schedules, improving mapping accuracy even under severe field variations, and compatible with multiple sequence types (e.g., standard MRF, CEST, or MT‐MRF) [76]. These strategies combine sequence selection (e.g., GRE vs. bSSFP), phase cycling, and inhomogeneity modeling or correction to ensure that physiological parameter estimation remains reliable despite magnetic field non‐uniformity for the clinical translation of physiological MRF.

Equally important is optimizing SNR, especially in MRvF applications. Wheeler et al. conducted simulations with varying SNRs to evaluate the accuracy of dynamic MRvF measures [77]. Their study showed that accurate vascular parameter matching in brain MRvF necessitates an SNR of at least 20. At this threshold, parameters such as SO_2_, CBV, and R demonstrated an RMSE reduction compared to lower SNR levels. Similarly, Boux et al. evaluated noise resilience in MRvF using synthetic and acquired signals [49]. They introduced Gaussian noise with SNRs of up to 60 and found that their Bayesian Dictionary‐Based Statistical Learning (DB‐SL) method outperformed traditional matching approaches. For synthetic vascular signals, DB‐SL achieved an RMSE of 1.87% for CBV and 5.78 μm for VSI at SNRs greater than 30, showcasing its robustness under realistic noise conditions.

Despite its innovative framework, MRF‐ASL is inherently low in SNR, especially in white matter, due to the small (˜1%) perfusion tag signal difference and the relatively weak sensitivity of the randomized label/control time course to vascular parameters [24]. This SNR challenge is amplified in populations with slower perfusion. While conventional ASL can employ long labeling duration to boost signal in low‐flow states, MRF‐ASL utilizes a series of short, randomized labeling blocks. This design can limit the accumulation of tagged blood, particularly when flow velocities are low and arterial transit times are prolonged, as is common in aging and neurodegenerative disease. Wright et al. quantitatively demonstrated that MRF‐ASL sensitivity to CBF, BAT, CBV, and other vascular metrics was much smaller than its sensitivity to T_1_ or FA, such that those vascular parameters are particularly vulnerable to noise‐induced errors [23]. Similarly, parameter recovery in MRF‐VFD inherently hinges on balancing field inhomogeneity correction, SNR, contrast dosage, and temporal sampling, with currently no clearly defined SNR thresholds. For example, B_1_ inhomogeneity can introduce substantial bias in kinetic parameters like K_trans_ unless corrected properly [78]. As a result, vascular permeability metrics with low signal sensitivity remain susceptible to noise, mirroring the limitations observed in pure MRF‐ASL approaches.

### 6.2 Integration of Machine Learning for Fingerprint Matching

The foundation of any supervised learning model is its training data. For physiological MRF, the most common strategy is to generate a large training set (often millions of examples) through numerical simulation of the governing physical equations (e.g., the Bloch equations coupled with a physiological model). This approach provides voxel‐wise ground‐truth labels, but it risks creating a gap between simulation and in vivo signals, where a model trained on idealized data fails to generalize to noisy, artifact‐laden in vivo signals. To mitigate this, it is critical to augment the training data to reflect real‐world conditions. This includes adding realistic noise (e.g., Rician noise at SNRs matching the target scanner) and simulating common artifacts like off‐resonance effects or motion. An alternative, more robust strategy involves training on high‐fidelity experimental data from phantoms or even using in vivo data where a reference standard (e.g., from a separate, validated acquisition) is available, which helps the network implicitly learn to handle real‐world signal variations.

The choice of network architecture must be matched to the structure of the MRF data. For a direct mapping from a signal time‐course to a set of parameters, a simple and effective starting point is a fully connected network, or multi‐layer perceptron (MLP). This architecture has proven successful for MRF‐ASL reconstruction, where the network learns a direct regression from the fingerprint vector to parameters like CBF and BAT [26, 38]. However, for fingerprints where the temporal ordering and dependencies contain critical information, a recurrent neural network (RNN) is a more powerful choice. Architectures like LSTM networks are explicitly designed to process sequential data, making them well suited for capturing the complex temporal evolution of the signal in techniques like MRvF, as demonstrated by the MARVEL framework [50]. A more advanced concept is the use of physics‐informed neural networks (PINNs), which embed the physical model directly into the network loss function [79]. This acts as a strong regularizer, compelling the network output to adhere to the laws of physics and potentially improving generalizability.

**FIGURE 8:**
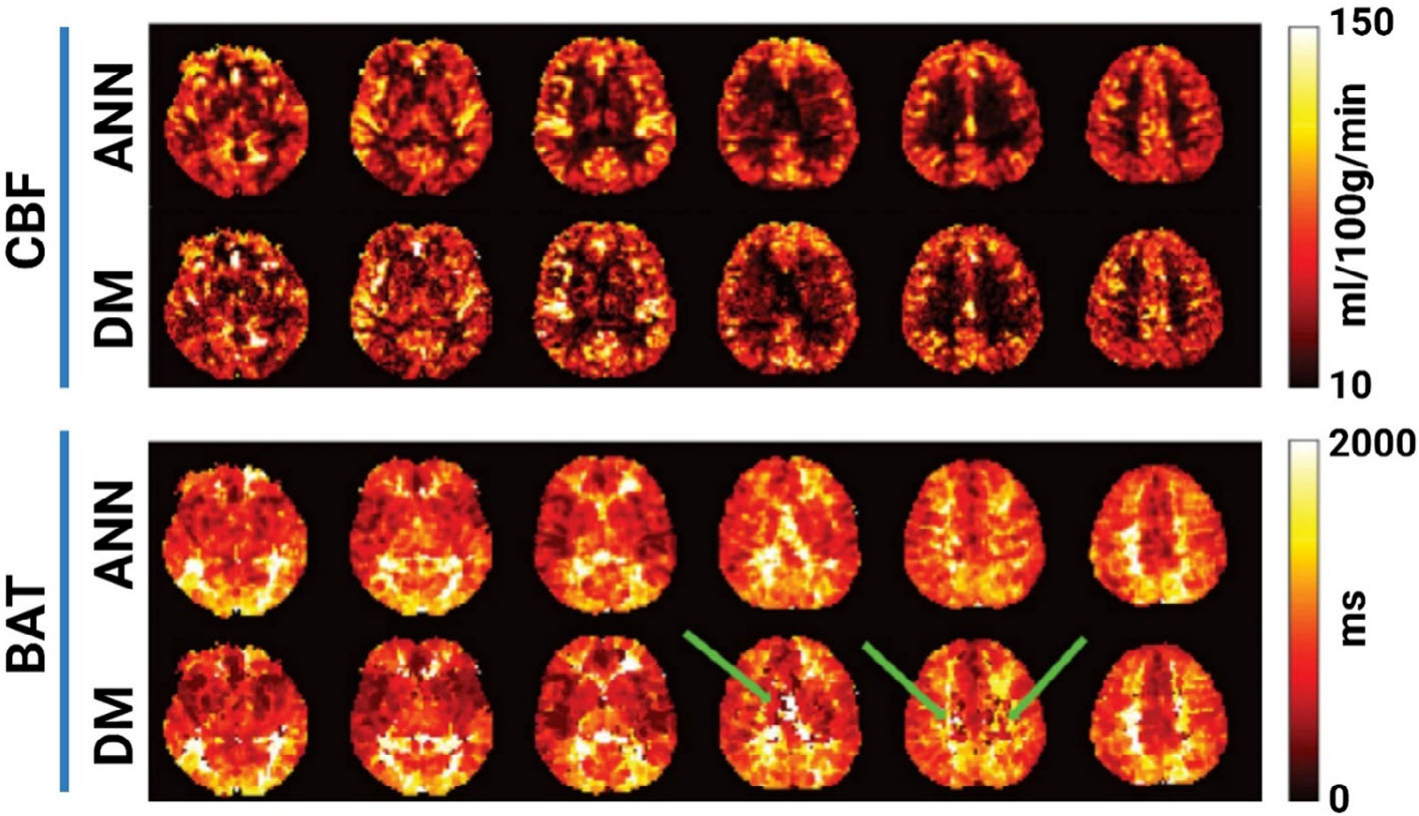
Comparison of parametric maps derived from ANN and DM in MRF‐ASL. The top row displays CBF maps, while the bottom row presents BAT maps. Each column represents data processed with DM and ANN, highlighting marked reductions in noise and artifacts in ANN‐derived maps. Green arrows in BAT maps point to regions of artifact correction achieved by the ANN. Adapted from Fan et al. [26], *Magn. Reson. Med*. 2020.

Deep learning significantly accelerates reconstruction times for physiological MRF imaging compared to traditional DM methods. The DeepMARS approach reduced reconstruction time per voxel to less than 0.5 milliseconds, compared to over 4 s per voxel with DM, translating to a 480‐fold improvement in efficiency for single‐compartment ASL models [38]. Similarly, Fan et al. (Figure 8) demonstrated that an ANN reduced total processing time for MRF‐ASL data from 3 h and 12 min with DM to just 3.6 s, achieving a 3000‐fold speed‐up [26]. These advancements not only enable real‐time or near‐real‐time processing but also maintain or improve the accuracy of parameter maps, making deep learning reconstruction advantageous for dynamic studies involving multiple time points or clinical applications requiring rapid hemodynamic mapping.

Finally, the “black box” nature of neural networks demands a rigorous validation strategy and an awareness of potential pitfalls [80, 81]. If a network encounters an in vivo signal that falls outside its training distribution (e.g., from a rare pathology or an unexpected artifact), it may still produce a plausible‐looking but physically meaningless result without an error flag. Therefore, validation must extend beyond simple metrics on a test set. It should involve assessing the model's performance on diverse datasets from different scanners and patient cohorts and comparing its output against established reference methods. It is also crucial to analyze the model's failure modes by testing it on data with known artifacts or extreme physiological values. Understanding these limitations is an essential prerequisite for the responsible translation of these powerful reconstruction techniques into clinical practice.

### 6.3 Challenges and Future Prospects

A central vision for advancing physiological MRF is to identify those clinical or research domains in which conventional methods fall short and where physiological MRF offers uniquely transformative potential. For example, non‐invasive, truly quantitative assessment of cerebral perfusion, oxygen metabolism, and BBB permeability in ischemic stroke, multiple sclerosis, brain tumors, and mild traumatic brain injury could be enabled by physiological MRF [82, 83]. These quantifications could support early diagnosis, treatment monitoring, and differentiation of overlapping pathologies in a single scan. Identifying and articulating these high‐impact use cases helps justify continued technical investment in physiological MRF and unify the many existing strategies.

The significant progress in physiological MRF motivates future innovation to address remaining challenges for wider application of these methods. Low SNR, motion artifacts, and the complexity of vascular dynamics complicate accurate parameter estimation [84]. Additionally, computational demands for dictionary generation and matching remain high, particularly for multi‐parameter studies involving intricate physiological models [85]. Addressing these limitations will require continued advancements in hardware, acquisition strategies, and computational techniques. Promising acquisition strategies, such as those using rotated or stack‐of‐spiral trajectories, can provide complementary spatial information that enables the reconstruction of high‐resolution datasets with enhanced SNR [86, 87].

Validation of new developments in physiological MRF is essential to ensure their accuracy and reliability for clinical translation. This process requires a multi‐tiered approach. The first step involves using standardized, static phantoms (e.g., the ISMRM/NIST MRI system phantom) to validate the fundamental accuracy and precision of the sequence relaxometry measurements (T_1_, T_2_) against known ground truths [88, 89]. However, for hemodynamic parameters such as CBF, CBV, and BAT, static phantoms are insufficient. Therefore, a crucial second step is validation against dynamic “flow phantoms” capable of mimicking perfusion and fluid exchange, which provides a controlled environment to test the underlying physiological models. The final step is rigorous in vivo validation, which involves correlating physiological MRF results with established reference imaging modalities like Positron Emission Tomography (PET) or other validated MRI techniques within the same scan session and on the same subjects to minimize physiological variability [90]. A major barrier to clinical translation is the lack of standardization across sites, scanners, and preprocessing pipelines. Harmonization efforts are critical to ensure reliable cross‐site comparisons and reproducibility of MRF‐derived imaging biomarkers. Existing frameworks from broader quantitative imaging such as the Brain/MINDS HARP protocol [91] and Quantitative Imaging Biomarker Alliance (QIBA) [92] guidelines provide useful templates for developing standardized protocols, quality‐control methods, and dictionary generation practices specific to physiological MRF.

A further limitation common to the physiological MRF methods is the dependence on a pre‐computed dictionary of simulated signal evolutions under assumed tissue properties [93]. These dictionaries are typically generated for healthy tissue models or simplified kinetic models and assume stable physiological processes and exchange/relaxation behavior. However, in pathological states, the actual magnetization evolution may diverge from the simulated fingerprints due to altered perfusion, permeability, microvascular orientation, inflow/outflow kinetics, and un‐modeled compartmental heterogeneity [94]. As a consequence, dictionary matching may yield misassignments, bias or reduced precision of parameter estimates when applied in disease conditions. It is therefore important that future work explore adaptive or pathology‐specific dictionaries, model augmentation (e.g., additional compartments, exchange modeling) and validation of fingerprinting approaches in diseased cohorts.

Future prospects for physiological MRF lie in expanding its clinical utility and integrating it with other modalities for a more holistic assessment. An exciting frontier for physiological MRF lies in its potential to probe the glymphatic system and to leverage advanced ASL contrasts, such as multi‐echo or ultra‐long TE ASL acquisitions, for enhanced insight into brain fluid dynamics. Recent ASL studies using ultra‐long TE and multi‐echo ASL have successfully quantified brain‐wide water transport from blood to cerebrospinal fluid in healthy humans. This work established a powerful method for directly quantifying glymphatic‐like activity, creating a pathway for future investigations into how these clearance dynamics are altered in various neurological disorders [95]. The integration of MRF into these advanced MRI techniques may improve the acquisition efficiency and shorten the scan duration to clinically practice time. Furthermore, the future integration of physiological MRF with simultaneous PET/MR allows for the concurrent, co‐registered acquisition of PET gold‐standard metabolic data (e.g., oxygen or glucose metabolism) with MRF high‐resolution quantitative maps of hemodynamics (CBF, CBV) and tissue properties. This powerful combination would enable a direct, voxel‐wise investigation of neurovascular coupling and tissue viability that is not possible when combining PET with conventional anatomical MRI alone [96, 97].

## 7 Conclusion

This review highlights the transformative potential of physiological MRF in advancing the understanding and assessment of cerebral physiology. By integrating advanced acquisition paradigms and sophisticated biophysical modeling, physiological MRF enables the simultaneous and non‐invasive quantification of critical physiological parameters. The adaptations of physiological MRF, such as MRF‐ASL, MRvF, and MRF‐VFD approaches demonstrate their versatility and applicability. Challenges such as low SNR, motion artifacts, and computational demands are being actively addressed through innovations like deep learning, which have shown promise in improving parameter mapping and accelerating dictionary‐based reconstructions. Future directions include expanding its clinical applicability to the diagnosis and monitoring of conditions such as stroke, brain tumors, and neurodegenerative diseases.

## Conflicts of Interest

The authors declare no conflicts of interest.

## Data Availability

Data sharing not applicable to this article as no datasets were generated or analyzed during the current study.
